# The Epstein Barr-encoded BART-6-3p microRNA affects regulation of cell growth and immuno response in Burkitt lymphoma

**DOI:** 10.1186/1750-9378-9-12

**Published:** 2014-04-14

**Authors:** Maria Raffaella Ambrosio, Mohsen Navari, Lorena Di Lisio, Eduardo Andres Leon, Anna Onnis, Sara Gazaneo, Lucia Mundo, Cristina Ulivieri, Gonzalo Gomez, Stefano Lazzi, Miguel Angel Piris, Lorenzo Leoncini, Giulia De Falco

**Affiliations:** 1 Department of Medical Biotechnologies, University of Siena, Via delle Scotte, 6-53100 Siena, Italy; 2 Department of Pathology, Hospital Universitario Marques de Valdecilla, IFIMAV, Santander, Spain; 3 Bioinformatics Unit (UBio), Structural Biology and Biocomputing Programme, Spanish National Cancer Research Centre (CNIO), Madrid, Spain; 4 Department of Life Sciences, University of Siena, Siena, Italy

**Keywords:** EBV, Burkitt lymphoma, MicroRNAs

## Abstract

**Background:**

Burkitt lymphoma is an aggressive B-cell lymphoma presenting in three clinical forms: endemic, sporadic and immunodeficiency-associated. More than 90% of endemic Burkitt lymphoma carry latent Epstein-Barr virus, whereas only 20% of sporadic Burkitt lymphoma are associated with Epstein-Barr infection. Although the Epstein-Barr virus is highly related with the endemic form, how and whether the virus participates in its pathogenesis remains to be fully elucidated. In particular, the virus may impair cellular gene expression by its own encoded microRNAs.

**Methods:**

Using microRNA profiling we compared Epstein-Barr-positive and Epstein-Barr-negative Burkitt lymphoma cases for both cellular and viral microRNAs. The array results were validated by qRT-PCR, and potential targets of viral microRNAs were then searched by bioinformatic predictions, and classified in functional categories, according to the Gene Ontology. Our findings were validated by *in vitro* functional studies and by immunohistochemistry on a larger series of cases.

**Results:**

We showed that a few cellular microRNAs are differentially expressed between Epstein-Barr-positive and Epstein-Barr-negative Burkitt lymphoma cases, and identified a subset of viral microRNAs expressed in Epstein-Barr-positive Burkitt lymphomas. Of these, we characterized the effects of viral BART6-3p on regulation of cellular genes. In particular, we analyzed the IL-6 receptor genes (*IL-6Rα* and *IL-6ST*), *PTEN* and *WT1* expression for their possible relevance to Burkitt lymphoma. By means of immunohistochemistry, we observed a down-regulation of the IL-6 receptor and PTEN specifically in Epstein-Barr-positive Burkitt lymphoma cases, which may result in the impairment of key cellular pathways and may contribute to malignant transformation. On the contrary, no differences were observed between Epstein-Barr-positive and Epstein-Barr-negative Burkitt lymphoma cases for WT1 expression.

**Conclusions:**

Our preliminary results point at an active role for the Epstein-Barr virus in Burkitt lymphomagenesis and suggest new possible mechanisms used by the virus in determining dysregulation of the host cell physiology.

## Introduction

Burkitt lymphoma (BL) has represented a key model for understanding multi-stage tumorigenesis [1]. It was the first tumor for which the association with an infectious agent was demonstrated [2]. The discovery in the 1960s of its association with the Epstein-Barr virus (EBV) became a foundation stone of human tumor virology [3]. Later, in the 1980s the identification of c-*MYC/Ig* fusion at the site of t(8;14) translocation has set a path that has opened the molecular basis of oncogenic changes in many other tumor context [4,5]. BL is now listed in the World Health Organization (WHO) classification of tumors of hematopoietic and lymphoid tissue as an aggressive B-cell non-Hodgkin’s lymphoma with three subsets, endemic (eBL), sporadic (sBL) and immunodeficiency-associated (ID-BL), which mainly differ for geographic distribution, clinical presentation and association with EBV [6].

More recently, focus has been restored on BL in a way that again highlights its potential in revealing new insights. Whole genome analysis elucidated the existence of common gene-coding mutations in BL [7].

Gene expression profile (GEP) has demonstrated that BL has a unique molecular profile, distinct from those of other B-cell non-Hodgkin lymphomas (B-NHLs) and, especially, from that of diffuse large B-cell lymphoma (DLBCL) [8,9]. In addition, we have described differences in terms of gene and microRNA expression among sBL and eBL [10,11]. eBL cases show an enrichment of genes involved in the B-cell receptor-signalling pathway, suggesting a direct role of chronic antigenic stimulation in Burkitt lymphomagenesis [10]. Most eBL are associated with EBV and occur in areas in which malaria is endemic, but there is still no satisfactory explanation of how and whether the virus participates in BL pathogenesis. EBV may impact on host cell homeostasis in various ways by interfering with cellular microRNAs (miRNAs) expression and by encoding its own genes and miRNAs; in fact we have recently demonstrated that EBNA1 is able to induce the expression of cellular miRNAs in BL [12]. The EBV genome encodes for 45 mature microRNAs from 25 precursors, which are mapped in 2 regions of the genome: BHRF1 (Bam HI fragment H rightward open reading frame I) and BART (Bam HI-A region rightward transcript) [13]. The BART region encodes the cluster 1 and cluster 2 EBV-miRNAs, whereas the BHRF1 region contains only 3 miRNAs [14]. EBV-encoded miRNAs are differentially expressed among the different latency programs, being the latency III restricted to BHRF1 miRNA expression and the latency I and II to BART miRNA expression [15]. However, to date, little information is still available about the expression of EBV-encoded miRNAs in primary tumours, and their possible contribution in dysregulating host cell gene expression [16-20].

With the aim to assess the contribution of EBV-encoded miRNAs in Burkitt lymphomagenesis, we profiled a number of EBV-positive and EBV-negative BL cases for cellular and viral miRNA expression. Then we validated our findings by *in vitro* functional studies and by immunohistochemistry on samples from a larger, well-characterized cohort of cases, with the attempt to shed light on new possible mechanisms used by the virus to contribute to malignant transformation and to favor the design of more specific treatments for EBV-associated malignancies. Our preliminary results point at an active role for the Epstein-Barr virus in Burkitt lymphomagenesis and suggest new possible mechanisms used by the virus in determining dysregulation of the host cell physiology.

## Methods

### Ethics statement

The study was approved by the Ethics Committee of Nairobi Hospital, CNIO Madrid, Spain and Siena University Hospital, Italy. Study participants or their legal guardians provided written informed consent.

### Cases selection

For this study 71 BL cases, collected at the Department of Pathology, Nairobi Hospital, Kenya, CNIO biobank, Madrid, Spain and at the Department of Medical Biotechnologies, University of Siena, Italy, have been used. Cases were reviewed by expert pathologists and diagnoses were confirmed according to the WHO [6]. Among the 40 eBL cases, 38 were EBV positive, whereas only 3 out the 31 sBL cases were EBV positive.

For miRNA profiling, 18 BL cases (six EBV-positive and twelve EBV-negative) have been used. Immunohistochemical studies were performed on representative sections of 35 EBV-positive and 18 EBV-negative formalin-fixed and paraffin embedded (FFPE) BL samples.

### EBER-in-situ hybridization (ISH) and EBV latency analysis

The presence of EBV was assessed by *in situ* hybridization for EBERs using Epstein-Barr Virus (EBER) PNA/Fluorescein (Dako, Milan-Italy), a mixture of PNA probes complementary to the two nuclear EBER RNAs encoded by EBV, in conjunction with Dako PNA ISH Detection Kit, according to manufacturer’s instructions. A control slide, prepared from a paraffin-embedded tissue block containing metastatic nasopharyngeal carcinoma in a lymph node, accompanied each hybridization run.

The expression of EBV-encoded genes (EBNA1, LMP1, EBNA2, Zebra), which characterize the different latency programs, has been investigated by qRT-PCR using Taqman probes, as previously described [21]. In addition, the expression of the relative proteins were checked by immunohistochemistry. Of these, BL cases expressed only EBNA1 at gene and protein level.

### MicroRNA extraction

Total RNA was extracted from FFPE sections of primary tumors and reactive lymph nodes using the PPPE miRNA Easy kit (Qiagen, CA), and from fresh tissues of eighteen cases of BL (six EBV-positive BLs and twelve EBV-negative BLs) was extracted using the miRNA Easy Kit (Qiagen, CA), according to the manufacturer’s instructions. The amount and quality of RNA were evaluated by measuring the optic density (OD) at 260 nm, the 260/230 and the 260/280 ratios using a Nanodrop spectrophotometer (ND-1000, Nanodrop, Thermo Scientific, Celbio-Italy). The cases were subjected to the Agilent® Human microRNA microarray technology (Agilent, Cernusco sul Naviglio-Italy) following manufacturer instructions.

### MicroRNA microarray expression profiling

Total RNA was extracted from fresh tissues of eighteen cases of BL (six EBV-positive BLs and twelve EBV-negative BLs) using TRIZOL reagent (Invitrogen, CA).

For miRNA detection, 100 ng of total RNA were hybridized on a one color Agilent Human miRNA Microarray (Agilent Technologies, Inc., Santa Clara, CA) following manufacturer instructions [22]. Scanning was carried out using the Microarray Scanner System (Agilent). Microarray images were processed using feature extraction software (Agilent technologies). Background subtraction and quantile normalization has been applied by using a script developed in collaboration with CNIO bioinformatics unit. To compare EBV-positive *versus* EBV-negative samples, a T-test that includes correction for multiple testing was performed using the web site: http://pomelo2.bioinfo.cnio.es/[23]. miRNAs showing FDR (False Discovery Rate; q-value) below the value of 0.05 were considered differentially expressed between the two groups. A heatmap of miRNAs, whose FDR was below 0.05, has been constructed using the web site: http://www.gepas.org. The microarray data has been deposited at the National Center for Biotechnology Information Gene Expression Omnibus database (accession number: GSE52916).

### Computational analysis and gene ontology

Target genes of the differentially expressed miRNAs isolated by profiling were identified by computational analysis, using web-available resources (Mirnaviewer, PicTar, Tarbase [24], miRBase) [25] and miRGate. miRGate is a tool developed in collaboration with bioinformatics unit at CNIO (Madrid). Target genes common to more than one prediction algorithm were considered. Functional studies were then performed on selected genes possibly of interest for BL. Genes were classified in functional categories according to the Gene Ontology [26].

### Real-time quantitative reverse transcription PCR (qRT-PCR)

Validation of both cellular (hsa-miR181d, hsa-miR609, hsa-miR574, hsa-miR197, hsa-miR142-5p, hsa-miR7, hsa-miR501-5p, hsa-miR510) and viral miRNAs, and the expression level of a selection of possible target genes, was obtained by RT-qPCR, using the specific Taqman microRNA Assays for cellular and viral miRNAs (Applied Biosystems, Germany), following manufacturer’s instructions. For target gene validation, reverse transcription was performed using the QuantiTect Reverse Transcription Kit (Qiagen, CA) and qPCR was made using the QuantiTect SYBR Green PCR Kit (Qiagen, CA), according to the manufacturer’s instructions. qRT-PCR was performed using a Rotorgene machine (Qiagen, CA). Exported result files were then loaded into Data Assist Software v2.0 (Applied Biosystems) for statistical analysis of differences in gene and miRNA expression. Normalization was made on RNU43, GAPDH and 18S rRNA which were stably expressed among the samples, and relative quantification was performed using the ΔΔCt method [27]. Statistical analysis was performed using the T-test [23].

### Immunohistochemistry

Representative sections (4-μm thick) were placed on positively charged glass slides (ProbeOn Plus; Fisher Scientific, Pittsburgh, PA, USA). The staining was carried out on Bond III automated immunostainer (Leica Microsystem, Bannockburn, IL, USA) and diaminobenzidine (DAB, Leica Microsystem, Bannockburn, IL, USA) was used as chromogen. A large panel of antibodies (See the list of stains) was applied and reactive lymph nodes were used as control. Expression level of both chains of Interleukin-6 (IL-6) receptor (p80 and gp130), PTEN and WT-1 protein was assessed using immunohistochemistry in 53 BL primary tumors (35 EBV-positive and 18 EBV-negative). A detailed list of the antibodies and working condition is reported in Table 1. Immunoreactivity was assessed by two blinded investigators and the expression levels were classified semiquantitatively combining the proportion and intensity of positively stained cells [28]. The percentage of positive-staining cells was scored as follows: 1 (<5% positive cells), 2 (5-50% positive cell), and 3 (>50% positive cells). Staining intensity was scored as follows: 1 (weak or not detectable staining), 2 (moderate staining) and 3 (strong staining). The sum of the staining intensity score and the percentage score was used to define the protein expression level, calculated as HSCORE [28]: 1, low expression; 2, moderate expression; 3, high expression. Differences in protein expression between EBV-positive and EBV-negative BL cases were analyzed using the Chi-square test. The level of statistical significance was established at p < 0.05.

**Table 1:** List of antibodies and their respective concentrations

**Primary antibody**	**Dilution**	**Company**
**CD20 (IHC)**	RTU	Novocastra- Leyca system, Milan (Italy)
**CD10 (IHC)**	RTU	Novocastra- Leyca system, Milan (Italy)
**Bcl-6 (IHC)**	RTU	Novocastra- Leyca system, Milan (Italy)
**Bcl-2 (IHC)**	RTU	Novocastra- Leyca system, Milan (Italy)
**CD38 (IHC)**	RTU	Novocastra- Leyca system, Milan (Italy)
**CD44 (IHC)**	RTU	Novocastra- Leyca system, Milan (Italy)
**IgM (IHC)**	1:100	Dako, Milan (Italy)
**Ki-67 (IHC)**	1:100	Novocastra- Leyca system, Milan (Italy)
**p80 (IHC)**	1:10	Abcam, Cambridge (UK)
**gp130 (IHC)**	1:50	Abcam, Cambridge (UK)
**PTEN (IHC)**	1:50	Dako, Milan (Italy)
**WT-1 (IHC)**	RTU	Novocastra- Leyca system, Milan (Italy)
**PTEN (WB)**	1:500	Dako, Milan (Italy)
**gp130 (WB)**	1:500	Abcam, Cambridge (UK)
**p80 (WB)**	1:500	Abcam, Cambridge (UK)
**phospho IκB-α (WB)**	1:1000	Cell Signaling, Monza, Italy
**Phosphor Akt (WB)**	1:500	Cell Signaling, Monza, Italy
**EBNA1 (WB)**	1:100	Santa Cruz Biotechnology, Santa Cruz, CA, USA
**Actin (WB)**	1:1000	Abcam, Cambridge, UK.

RTU: ready to use.

### Cell culture

BL-derived cell lines were used for *in vitro* studies. Briefly, Ramos (EBV-negative), Raji (EBV-positive) were obtained from the American Type Culture Collection (ATCC). The Akata cell line (EBV-positive) and its defective counterpart Akata 2A8 (which is an EBV-negative variant of the Akata cell line) were kindly provided by Prof. Trivedi (University La Sapienza, Rome, Italy). Ramos and Raji cells were cultured in RPMI 1640 medium supplemented with 10% Fetal Bovine Serum (FBS), 2 mM glutamine, 100U/ml penicillin and 100 μg/ml streptomycin (all from Lonza, Swiss). Akata cells were cultured in the same medium as Ramos cell line, supplemented with 1 mM sodium pyruvate and 100 nM MEM non-essential amino acids (both from Gibco-Life Technologies, Monza-Italy). For inhibition of the endogenous EBV-Bart6-3p, cells were grown in growth medium, as previously described before transfections. Proliferation rate was measured by Tripan blue staining.

### Cell transfection

Based on the highest level of expression of BART6-3p, *in vitro* studies using mimic and inhibitor sequences of BART6-3p were performed in Akata cells. Briefly, transient transfections of the Akata cell line were performed using an Amaxa nucleofector apparatus (Amaxa, Cologne-Germany), program G23 and transfection solution V according to the manufacturer’s instructions. 5×10^6^ cells were transfected with 100 nM of BART6-3p inhibitor (Custom synthesized by Dharmacon- Thermo Scientific, Germany), 10 nM of negative control of miRNA inhibitor (I-300145-01; Dharmacon-Thermo Scientific, Germany) or the transfection solution as a mock. Transfection efficiency was assessed using transfecting 2 μg of pmaxGFP and detecting both fluorescence and cell viability by flow cytometry. Cells were harvested 24 hours after transfection for RNA extraction and qRT-PCR and 48 hours post-transfection for protein extraction and western blotting.

### Western blotting

Western botting was performed as previously described [17]. Briefly, cells pellets were lysed on ice for in EBC buffer (50 nM Tris–HCl pH 8.0, 130 mM NaCl, 1% Triton X-100, 0.1% SDS) supplemented with protease inhibitor cocktail (Sigma, Milan-Italy). Cell lysates were separated by 10% SDS-PAGE gel followed by transfer to Hybond ECL nitrocellulose membrane (GE Healthcare, Milan, Italy). Secondary antibodies conjugated with HRP were used at a dilution of 1:5000 and the reaction was revealed using the ECL Western Blotting Kit (Promega, Milan-Italy) according to the manufacturer’s instructions. Antidody dilutions are reported in Table 1. Secondary antibodies conjugated with HRP were used at a dilution of 1:5000 and the reaction was revealed using the ECL Western Blotting Kit (W1001, Promega, Milan-Italy) according to the manufacturer’s instructions.

### Detection of apoptosis

Apoptosis was analyzed by flow cytometric analysis of cells stained with FITC-conjugated Annexin V and 50 μg/ml PI using Annexin V apoptosis Detection kit FITC (eBioscence). Samples were analyzed in a FACScan flow cytometer (Becton Dickinson, San Jose, CA) using CellQuest software. Doublets were excluded and 20.000 events were acquired for each sample.

## Results

### Epstein Barr-positive Burkitt lymphoma and Epstein Barr-negative Burkitt lymphoma differ for both cellular and viral miRNAs

**Figure 1:**
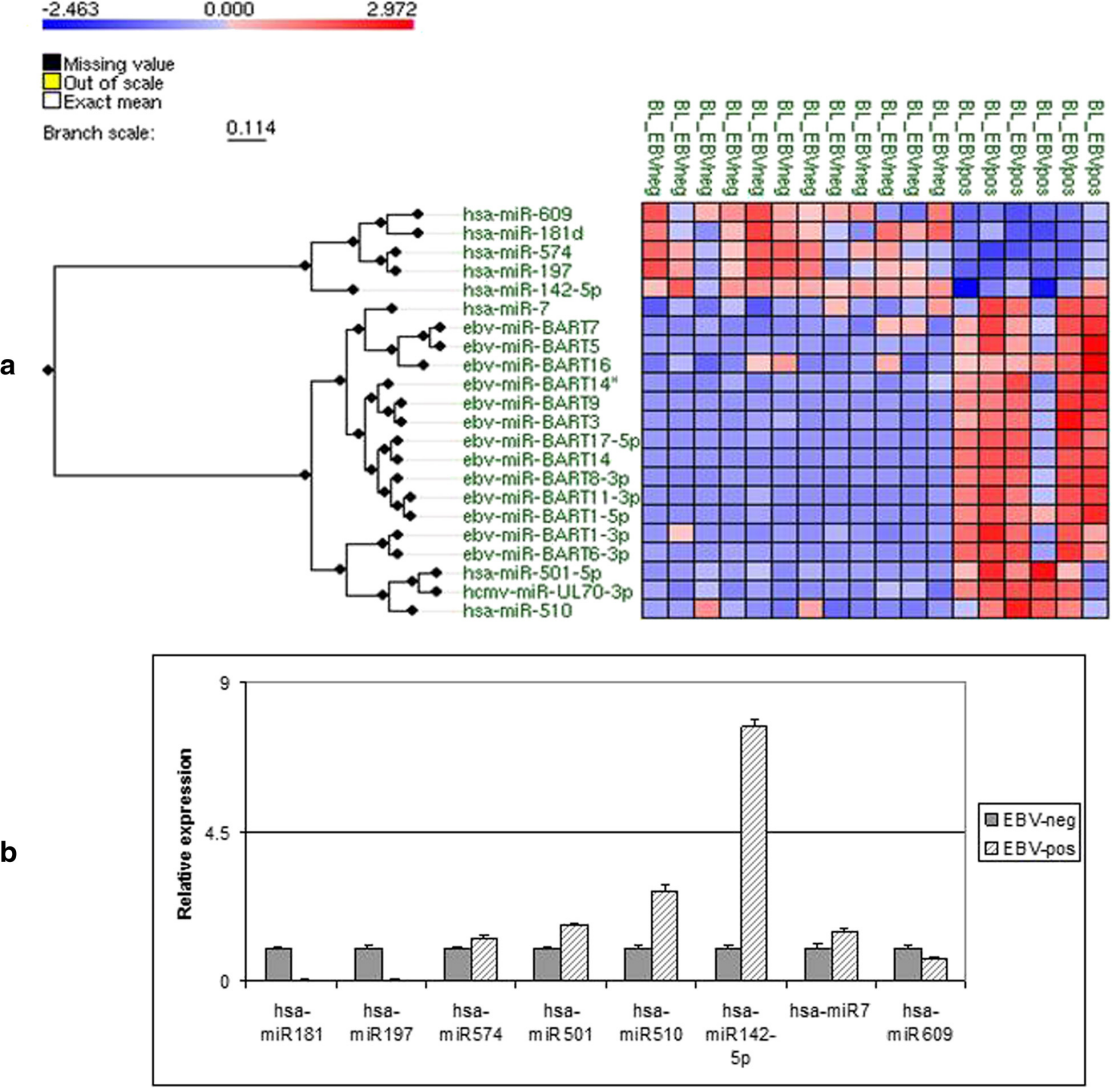
**microRNA profiling of EBV-positive vs. EBV-negative BL cases. (a)** Heatmap of miRNA differential expression in EBV-positive BL versus EBV-negative BL cases. 12 EBV-negative and 6 EBV-positive BL cases were compared in terms of viral and human miRNA expression using the Agilent® microRNA expression microarray technology. While each column represents a BL case, rows represent human miRNAs differentially expressed in two groups or viral miRNAs expressed in EBV-positive cases. We recognized 8 out of 470 cellular miRNA deregulated in EBV-positive and -negative BLs (FDR < 0.05). Indeed 13 out of 46 EBV miRNAs were found to be expressed in EBV-positive BL cases. **(b)** Validation by RT-qPCR of the array results on the cellular miRNAs differentially expressed between EBV-positive and EBV-negative BL cases. The graph is representative of three different qRT-PCR experiments. Error bars represent standard deviation between duplicates.

With the aim of identifying the EBV-encoded miRNAs expressed in BL primary tumors, we performed a microRNA profiling comparing EBV-positive *versus* EBV-negative BL cases, using a platform containing both cellular and viral miRNAs. The profiling identified 8 out of 470 cellular miRNAs, which were differentially expressed between EBV-positive and EBV-negative BLs (FDR < 0.05). In particular, 3 of these were up-regulated (hsa-miR-7, hsa-miR-501-5p, hsa-miR-510) and 5 miRNAs were down-regulated (hsa-miR-181d, hsa-miR-609, hsa-miR-574, hsa-miR-197, hsa-miR142-5b) in EBV-positive BL cases (Figure 1a). In addition, 13 out of 45 EBV miRNAs were found to be expressed in EBV-positive BL cases, all of which were from the BART coding region, including both BART cluster 1 and BART cluster 2 (Figure 1a). None of BHRF coding region miRNAs was found to be expressed in EBV-positive BL cases, confirming that BHRF miRNAs are not expressed in latency I. Validation and confirmation of the array results on cellular miRNAs was performed by qRT-PCR (Figure 1b).

### Key cellular pathways may be affected by dysregulated cellular and viral miRNAs

Potential target genes of cellular and viral dysregulated miRNAs were identified by prediction algorithms, and then subsequently classified in functional categories, according to the Gene Ontology [26]. This analysis revealed their involvement in cell cycle, proliferation, signal transduction, apoptosis and differentiation (Table 2).

**Table 2:** Target genes functional categories

**Protein binding**	**Cell proliferation**	**Signal transduction**	**DNA binding**	**Receptor activity**
AKAP9	PTEN	CXCL12	IRF5	TLR4
CALM2	CD81	IL-6 receptor		
MYRIP	WT1	MAPK12		
NFkBIE				
PEX5				
PTN7				
CASP2				
INPP5D				
CHUK				
SOD2				

Based on these predictions, we focused on BART6-3p, as it is supposed to regulate the expression of cellular genes involved in relevant cellular pathways, including tumor suppressors and signal transducers, whose imbalance may crucially contribute to malignant transformation (Table 3).

**Table 3:** Target genes of BART6-3p and their respective functions

**Target genes**	**Functional categories**
AKAP9	Protein binding
CALM2	Protein binding
CD81	Cell proliferation
CHUK	Protein binding
CXCL12	Signal transduction
p80	Signal transduction
gp130	Signal transduction
INNPP5D	Protein binding
MAPK12	Signal transduction
PTEN	Cell proliferation
NFKB1E	Protein binding
PEX5	Protein binding
PTPN7	Protein binding
WT1	Cell proliferation

**Figure 2:**
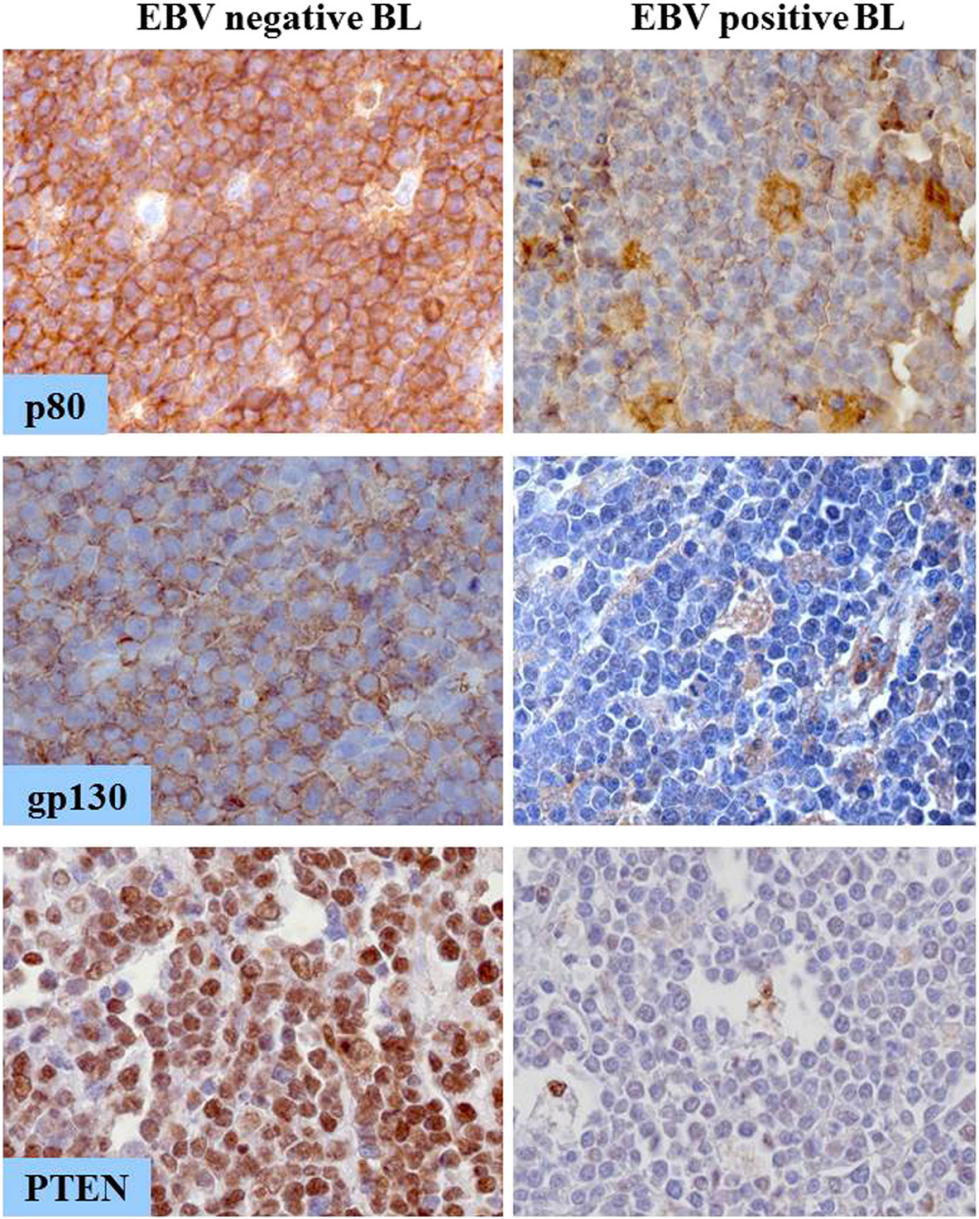
**Immunohistochemistry of BART6-3p target genes.** The expression level of p80, gp130 and PTEN was evaluated in EBV-positive (right) and EBV-negative (left) BL cases. p80 was strongly expressed in the cytoplasm (upper panel), gp130 showed membrane and cytoplasmic positivity (middle panel), PTEN was found in the nucleus (lower panel). Macrophages were used as internal positive controls. p80, gp130 and PTEN were found to be meaningfully down-regulated in EBV positive BL cases (right panel, upper, middle and lower images, respectively). (400x magnification).

In particular, BART6-3p is predicted to regulate the expression of *PTEN*, *WT1* and of both chains of IL-6 receptor (p80 and gp130), which are involved in key cellular functions such as proliferation, apoptosis, and immune surveillance. The expression of these genes was therefore analyzed by immunohistochemistry in additional 35 EBV-positive and 18 EBV-negative BL primary tumors. Both chains of IL-6 receptor and PTEN expression significantly differed between EBV-positive and EBV-negative cases, being both proteins strongly down-regulated in the former. p80 showed a cytoplasmic pattern with a membrane reinforcement whereas gp130 displayed a membrane and somewhat cytoplasmic expression. PTEN exhibited a strong nuclear positivity. Higher p80, gp130 and PTEN expression was observed in EBV-negative BL cases in comparison with EBV-positive BL cases (p < 0.001). The correlation between p80 and gp130, and PTEN expression in all BL samples is given in Tables 4, 5 and 6. In the EBV-positive cases showing IL-6 receptor and PTEN expression, the positivity was mild. Macrophages served as internal positive control for all the antibodies (Figure 2). On the contrary, no differences were appreciated between EBV-positive and EBV-negative BL cases for WT-1, that resulted not expressed in all of 53 cases (data not shown).

**Table 4:** Correlation between p80 expression and EBV status

	**BL EBV-positive cases**	**BL EBV-negative cases**	**Total (n.)**
**p80 positive cases (n.)**	2	17	19
**p80 negative cases (n.)**	33	1	34
**Total (n.)**	35	18	53

BL: Burkitt lymphoma; n.: number of cases.

**Table 5:** Correlation between gp130 expression and EBV status

	**BL EBV-positive cases**	**BL EBV-negative cases**	**Total (n.)**
**gp130 positive cases (n.)**	4	15	19
**gp130 negative cases (n.)**	31	3	34
**Total (n.)**	35	18	53

BL: Burkitt lymphoma; n.: number of cases.

**Table 6:** Correlation between PTEN expression and EBV status

	**BL EBV-positive cases**	**BL EBV-negative cases**	**Total (n.)**
**PTEN positive cases (n.)**	2	17	19
**PTEN negative cases (n.)**	33	1	34
**Total (n.)**	35	18	53

BL: Burkitt lymphoma; n.: number of cases.

### BART6-3p modulates the expression of IL-6 receptor and PTEN in vitro

To confirm the possible regulation by BART6-3p on the expression of the IL-6 receptor and PTEN, we performed a series of *in vitro* experiments using BL-derived cell lines, expressing the same latency program as BL primary tumors. First of all, we analyzed several EBV-positive BL-derived cell lines for the expression of BART6-3p, to select an appropriate cell model for functional studies.

**Figure 3:**
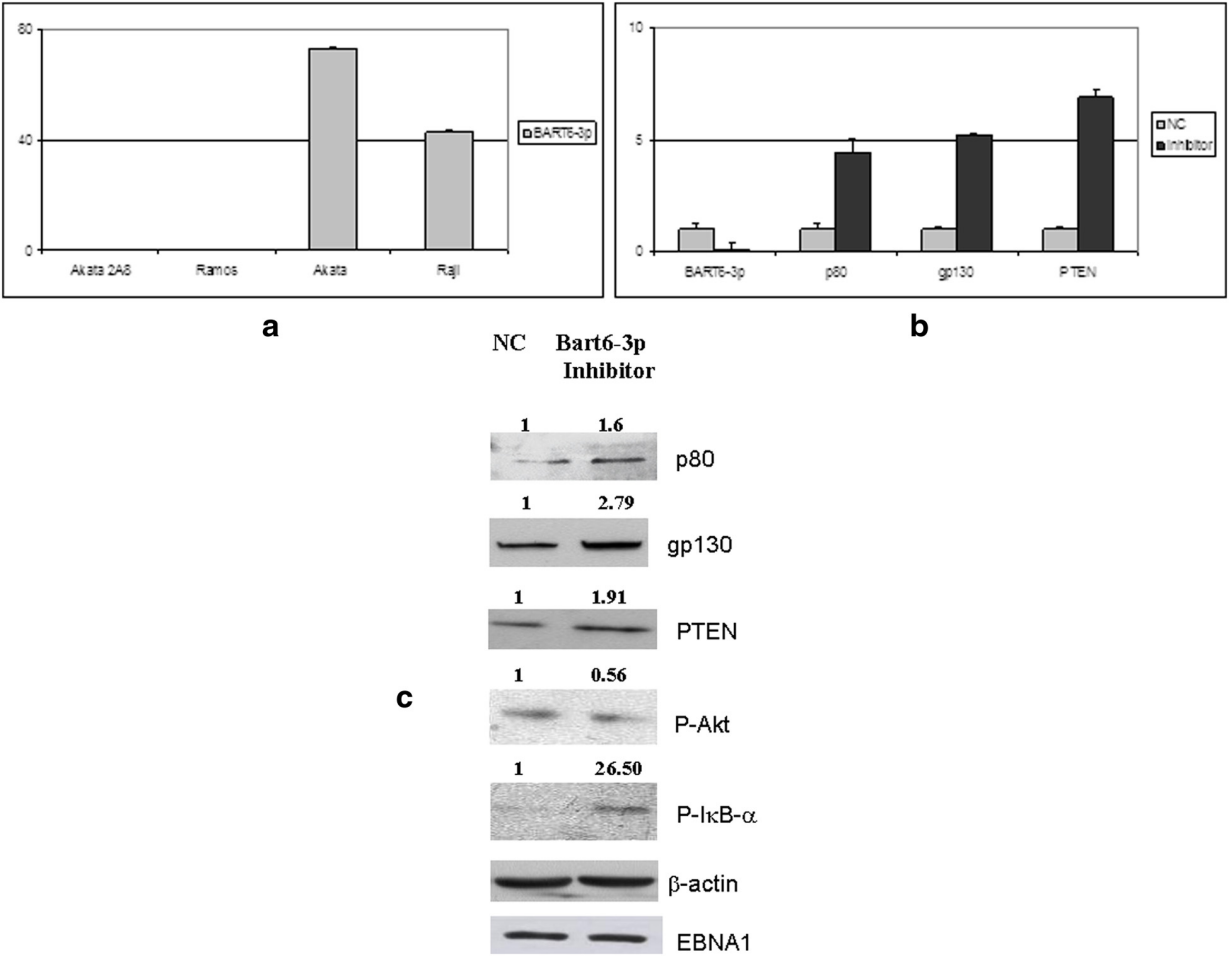
**BART6-3p-regulated pathways in BL-derived cell lines. (a)** The expression of BART6-3p was evaluated in two EBV-negative BL-derived cell lines (Akata 2A8 and Ramos) and two EBV-positive BL-derived cell lines (Akata and Raji), to select that expressing BART6-3p at the highest level. The Akata cell line shows higher expression level of BART6-3p. **(b)**: qRT-PCR of BART6-3p target genes following BART6-3p inhibition. A BART6-3p antagomir was transfected in Akata cells and the expression level of p80, gp130 and PTEN was evaluated 24 hrs post-transfection. Cells transfected with BART6-3p inhibitor showed a marked up-regulation of p80, gp130 and PTEN, in respect with cells transfected with the negative control (NC), confirming regulation of these genes by BART6-3p. As a control, down-regulation of the endogenous BART6-3p was observed following transfection with the antagomir. The graph is representative of three different qRT-PCR experiments. Error bars represent standard deviation between duplicates. **(c)**: Inhibition of BART6-3p by its antagomir results in the activation of the downstream pathways of the IL-6 receptor (p80 and gp130) and PTEN. The expression of p80, gp130 and PTEN was evaluated at the protein level by WB. In addition, two downstream signaling pathways were also evaluated following BART6-3p inhibition, the activation of NF-κB, which occurs through IκBα phosphorylation, and the decrease of the phosphorylated form of Akt, which acts downstream from PTEN. Protein levels were evaluated 48 hours post-transfection by Western blotting. The bands were then analyzed by the Image J software and relative expression levels were normalized to those of β-actin. Activation of NF-κB is consequent to IκB-α phosphorylation; PTEN activity results in the negative regulation of Akt, documented by a lower phosphorylation level. EBNA1 expression was measured as a control for EBV-positivity. The figure is representative of three different experiments.

Our results indicated the Akata cell line as the most suitable cell model, because of the high endogenous levels of BART6-3p (Figure 3a).

We then monitored whether the expression of the selected target genes could be modulated upon inhibition of the endogenous BART6-3p, in the Akata cell line. Therefore, to confirm that the differential expression of these genes was specifically dependent on BART6-3p, we ectopically modulated the expression of the endogenous BART6-3p in the Akata cells, by transiently transfecting a synthetic BART6-3p inhibitor.

The expression level of both subunits of the IL-6 receptor and PTEN was then checked by qRT-PCR 24 hrs after transfection; inhibition of BART6-3p was also evaluated, as a control. As expected, inhibition of the endogenous BART6-3p was achieved, and this resulted in the up-regulation of both chains of the IL-6 receptor and PTEN, both at the mRNA (Figure 3b) and protein levels (Figure 3c).

We then evaluated whether the up-regulation of these genes, consequent to BART6-3p inhibition, may affect their respective downstream pathways. Following binding of IL-6 with its receptor, a downstream signaling is transduced through the activation of NF-κB. This requires cleavage of NF-κB, which occurs through phosphorylation and consequent degradation of its inhibitor IκB-α. To determine whether IL-6 receptor up-regulation resulted in NF-κB activation, we measured the levels of phosphorylation of its inhibitor IκB-α.

The up-regulation of the IL-6 receptor, resulting from BART6-3p inhibition, leads in turn to the activation of NF-κB signaling, as demonstrated by increased phosphorylation of IκB-α. On the other hand, PTEN negatively regulates the activation of Akt, which occurs through phosphorylation of the Akt protein. Activation of PTEN, following BART6-3p inhibition, results in a decrease of the Akt phosphorylation and consequent Akt activity (Figure 3c).

### BART6-3p influences cell proliferation and cell death

We investigated whether this viral microRNA could have an effect in terms of cell viability. The effects of BART6-3p on cell proliferation were monitored upon inhibition of the endogenous viral microRNA by an antagomir.

**Figure 4:**
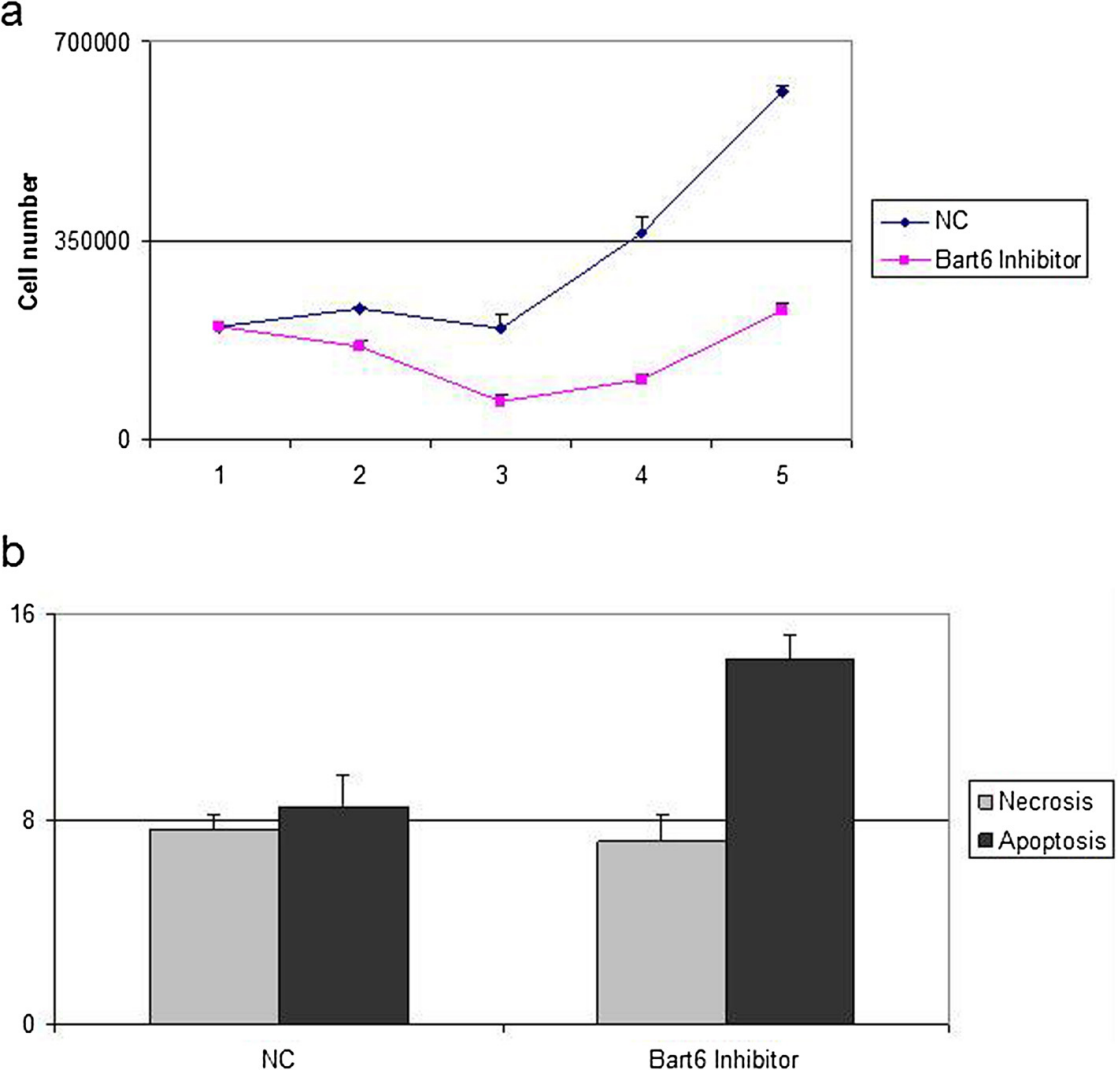
**Effects of BART6-3p on cell viability. (a)** The effects of BART6-3p on cell proliferation and cell death were monitored following its inhibition by a synthetic antagomir, by Tripan blue exclusion assay. Proliferation curves show that inhibition of BART6-3p results in a decreased proliferation rate, whereas Tripan blue staining revealed an increased in the dead cell fraction (data not shown). **(b)** To detect whether cell death could be dependent on apoptosis, an Annexin V staining was performed 24 hrs after inhibitor transfection. Our results indicate an increase of the apoptotic fraction of cells transfected with BART6-3p inhibitor, in respect with its negative control (NC). The figure is representative of three different experiments.

Silencing BART6-3p resulted in a decrease of the proliferation rate (Figure 4a); in addition, our results showed that cells transfected with BART-6-3p inhibitor had a higher mortality rate, when compared to negative control, measured by Tripan blue staining (data not shown). To specifically detect whether cell death could be due to induction of apoptosis, we performed Annexin V staining on cells transfected with the inhibitor of BART6-3p. Our results demonstrated that inhibition of BART6-3p resulted in an increase of apoptosis (Figure 4b).

## Discussion

Since its discovery as the first human tumor virus, EBV has been implicated in the development of a wide range of B-cell lymphoproliferative disorders, the first being BL [29-31]. However, the exact mechanism by which EBV promotes oncogenesis is still matter of discussion [32-34]. EBV may contribute to transformation through its encoded genes and/or miRNAs [35,36]. There is increasing evidence of a complex interplay between viruses and the cellular miRNA machinery, with viruses that encode their own miRNAs and viruses that actually employ cellular miRNAs as co-factors for replication [20]. EBV-encoded miRNAs may compete with cellular miRNAs and target cellular genes, thus dysregulating cellular pathways [35]. Therefore, the EBV-encoded miRNAs dramatically increase the complexity of potentially biologically active molecules produced by EBV during latent infection and could also have consequences on the pathogenicity of the infection [37,38].

To address a better knowledge of the role of EBV in BL, in this study we have compared EBV-positive *versus* EBV-negative BL cases by miRNA expression profile. Interestingly, the platform we used included both cellular and viral miRNAs, to highlight the contribution of EBV-encoded miRNAs in regulating host cellular gene and miRNA expression.

Our results identified few cellular miRNAs differentially expressed between EBV-positive and EBV-negative BL, being in line with recent studies, although different platforms and different techniques may be responsible for subtle differences [10,18,19]. Among the differentially expressed cellular miRNAs, hsa-miR-197 was found to be specifically down-regulated in EBV-positive BL tumors. This finding may be of interest, as this miRNA is predicted to regulate *BCL6*, which is a key regulator of germinal center exit and differentiation towards plasma cells. Previous studies from our group report that B-cell differentiation is impaired in EBV-positive BL due to the combinatory expression of hsa-miR-127 and EBNA1 [11,18]. Therefore, hsa-miR197 down-regulation may add complexity to the regulatory network of B-cell differentiation in BL.

We focused on EBV-encoded miRNAs, as their possible relevance for Burkitt lymphomagenesis has been poorly explored. Interestingly, we observed that all of the viral miRNAs expressed in EBV-positive BL cases are from the BART coding region, both cluster 1 and cluster 2, whereas no miRNAs from the BHRF regions were expressed. The absence of BHRF1 miRNAs from EBV-associated tumors is consistent with the latency I program expressed by BL, confirming previous reports on EBV-encoded miRNA expression in EBV-associated tumors [14]. Among all the BART-derived miRNAs, in this paper we focused on BART-6-3p, as it is predicted to target several cellular genes involved in cell cycle, cell proliferation, apoptosis, signal transduction, differentiation. Dysregulation of these pathways may crucially affect key cellular homeostasis. In particular, we evaluated the expression of *PTEN*, *WT1* and *IL-6* receptor (p80, gp130) genes, as they act as tumor suppressors and signal transducers. A marked down-regulation of PTEN and IL-6 receptor in EBV-positive cases was demonstrated. To confirm that BART6-3p regulates the expression of these genes, functional *in vitro* studies were carried out revealing that modulation of BART6-3p expression is inversely related to that of *PTEN* and *IL-6* receptor subunits. These results were also confirmed by analyzing PTEN and IL-6 receptor downstream genes.

PTEN functions as a tumor suppressor by negatively regulating the Akt/PI3K signaling pathway, which is overactive if PTEN is faulty or deficient, thus reducing apoptosis and allowing proliferation by induction of mTOR [39]. Impairment of PTEN may result in a growth advantage of infected cells, which may eventually contribute to malignant transformation [40]. In line with this observation, we found that re-expression of PTEN following BART6-3p inhibition results in increase of apoptosis, and in a reduction of the proliferation rate, which is in line with PTEN negative regulatory function.

The IL-6 receptor is a heterodimeric receptor, composed by the subunit alpha (p80), which is specific for IL-6 binding, and the beta chain (gp130), which is the common transducer of different cytokines belonging to the IL-6 family [41]. The IL-6 receptor regulates key cellular processes as cell proliferation, cell survival, and response to host pathogens following the binding of the dimerized receptor with its specific cytokine (IFN-α, IL-12, IL-27) [41,42]. Interestingly, a recent study has shown that gp130 is down-regulated in EBV-positive nasopharyngeal carcinoma primary tumors and determines the lack of activation of the Jak2/Stat pathway following IL-27 release, resulting in an impairment of NK cell function and immune surveillance [43]. Here we give evidence for the first time of the down-regulation of the IL-6 receptor also in EBV-positive BL primary tumors and derived cell lines. Our findings suggest that this may be due to BART6-3p through either direct and indirect mechanisms, providing room for additional functional effects of EBV in evading the immune response and in contributing to the tumorigenesis [44-46].

Collectively, our data confirmed that different pathogenetic mechanisms may exist in EBV-positive and EBV-negative BL tumors, and that EBV may have a driving role in Burkitt lymphomagenesis. This may be achieved through dysregulation of key cellular pathways by viral-encoded miRNAs. In particular, EBV-BART-6-3p may play an important role in the pathogenesis of BL, as it may affect the function of important signal transducers as NF-κB and Akt/PI3K [47]. In addition, it may interfere with innate and adaptive immune response, contributing to immune evasion of infected BL cells, and may impact on cell proliferation, cell growth and apoptosis by down-regulating PTEN, thus removing the inhibitory brake on cell proliferation.

## Conclusions

Despite the encouraging results on viral miRNAs, future experiments are needed. Nevertheless, our study may help to clarify the complex regulatory network between host and pathogen, and may serve as a paradigm for all the virus-related neoplasms in which pathogenic mechanisms remain still poor understood.

## Abbreviations

BART: Bam HI-A region rightward transcript; BHRF1: Bam HI fragment H rightward open reading frame I; BL: Burkitt lymphoma; eBL: Endemic Burkitt lymphoma; sBL: Sporadic Burkitt lymphoma; ID-BL: Immunodeficiency-associated Burkitt lymphoma; B-NHL: B-cell non Hodgkin lymphoma; DAB: Diaminobenzidine; DLBCL: Diffuse large B-cell lymphoma; EBV: Epstein-Barr virus; GEP: Gene expression profiling; FFPE: Formalin-fixed and paraffin embedded; IL-6: Interleukin-6; miRNAs: Micro RNAs; OD: Optic density; qRT-PCR: Real-time quantitative reverse transcription PCR; WHO: World Health Organization.

## Competing interests

The authors declare that they have no competing interests.

## Authors’ contributions

GDF, LL and MAP conceived and designed the experiment; GDF, MRA and LL draft the paper; MN, LDL, AO, GG, SG, LM and CU performed the experiment; MRA, EAL and SL analyzed the data; EAL and LDL contributed reagents/materials/analysis tool. All authors read and approved the final manuscript.
